# Primary sarcoma of the prostate: A case series of 6 patients

**DOI:** 10.1097/MD.0000000000042388

**Published:** 2025-05-09

**Authors:** Ping Yang, Li Wang, Yang He

**Affiliations:** a Department of Oncology, Shanghai Eighth People’s Hospital, Shanghai, China; b Department of Urology, Liyang People’s Hospital, Liyang, Jiangsu, China; c Department of Interventional Oncology, Dahua Hospital, Shanghai, China.

**Keywords:** case report, clinical features, poor prognosis, primary prostate sarcoma

## Abstract

**Rationale:**

Primary prostate sarcomas are highly malignant tumors characterized by their low incidence and poor prognosis. Due to this rarity, we present a limited number of case series.

**Patient concerns:**

In this report, we detail the clinical diagnosis, treatment, and prognosis of the last 6 primary prostatic sarcoma cases.

**Diagnoses:**

The 6 patients, all adults aged between 21 and 51 years, were diagnosed pathologically through needle biopsy or surgical specimens.

**Interventions:**

All patients underwent chemotherapy.

**Outcomes:**

At the time of the last follow-up, only 1 patient continued treatment, while the other 5 had succumbed to disease progression. The overall survival time ranged from 3 to 46 months.

**Lessons:**

Primary prostatic sarcoma presents with an insidious onset and rapid progression, lacking standard treatment options and associated with a very poor prognosis. Early detection and timely surgical intervention may improve the cure rate and extend survival time for affected patients.

## 1. Introduction

Prostate sarcoma is an uncommon malignancy, representing approximately 0.1% to 0.7% of all prostate cancer cases since the prostate mainly consists of glandular tissue, with adenocarcinomas being the most malignant tumors.^[1,2]^ Only around 5% of all soft tissue sarcomas affect the genitourinary tract.^[3]^ It predominantly affects adults aged 37 to 50 years, which is significantly younger than the average age at which prostate cancer is diagnosed. The primary symptom reported is lower urinary tract obstruction, typically resulting from prostate enlargement. While surgical excision is the standard treatment, the prognosis remains exceedingly poor in cases where metastasis is present. Here we describe the clinicopathological features of 6 cases of primary sarcoma of the prostate with 4 different pathological types, 5 cases of which have poor prognosis.

## 2. Case series

**Figure 1.**
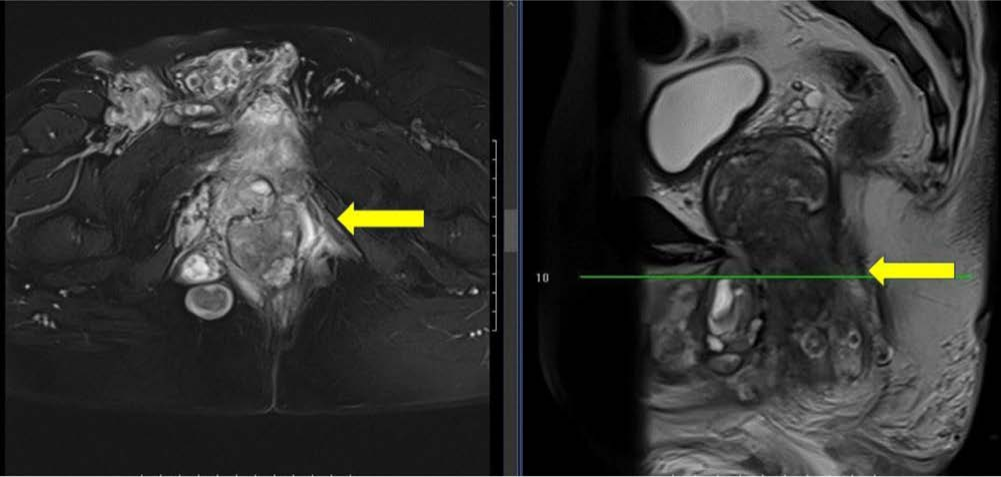
Pelvic MRI of Case 2. MRI = magnetic resonance imaging.

Six patients with an age rang from 21 to 51 years. Among the 6 patients, the pathologies of 3 cases were identified as completely embryonic rhabdomyosarcoma (Case 2, Case 4, and Case 5). All 6 patients experienced varying degrees of urinary difficulty at the time of diagnosis. Notably, when Case 6 was first diagnosed, nuclear magnetic resonance imaging revealed that the maximum diameter of the prostate tumor was 7.6 cm (Fig. 1), while the tumor sizes of the other 5 patients ranged from 4.3 to 5.6 cm. No local or distant lymph node metastasis was identified in any of the 6 patients. However, all 6 patients presented with distant metastasis at the time of diagnosis. Case 1 exhibited metastases to the lung, liver, and multiple bones. Cases 5 and 6 all showed pelvic metastasis, Case 2 demonstrating rectal infiltration (Table 1).

**Table 1:** Clinical features and overall survival time of 6 primary prostate patients.

	Case number	1	2	3	4	5	6
	Age (yr)	40	51	31	28	33	21
TNM	Tumor size (cm)	5	4.3	6	5.6	4.7	7.6
	Lymph node state	Free	Free	Free	Free	Free	Free
	Distant metastasis at the time of diagnosis	Lung Multiple bone Liver	Rectum Pelvic cavity	No	No	Pelvic cavity	Pelvic cavity
Pathologic results	Pathologic type	Triton tumor	Completely embryonic rhabdomyosarcoma	Spindle cell sarcoma	Completely embryonic rhabdomyosarcoma	Completely embryonic rhabdomyosarcoma	Synoviosarcoma
	Vascular cancer embolus	Positive	No submitted	Positive	No submitted	Positive	No submitted
	Immunohistochemistry	Desmin+, MyoD1 weak+, S-100 focal+, SMA focal+, PR focal+	Desmin+, Myogenin+, MyoD1 weak+, SMA−, ALK locally+, S-100−	SS18-SSX−, AE1/AE3-, CD34-, SMA−, Desmin−, H3K27M3+, vimentin+, STAT6-	Desmin+, MyoD1+, Myogenin+, ALK−, SMA−, S-100−, Caldesmon−, CD34−, NTrk− , CD117-DOG1−, SS18-SSX−	Desmin partially+, Myogenin partially+, WT1plasma+, CD56+, SMA partially+	CK+, EMA+, Vimentin+, Bcl-2+, CD99+, S-100 partially+, SMA−, Desmin−, CD34−, SYT-SSX+
	Ki-67 (%)	70	70	80	75	85	70
Treatment	Surgery	No	Yes	Yes	Yes	Yes	No
	Themotherapy	Doxorubicin Ifosfamide Dacarbazine	Epirubicin Ifosfamide	Vincristine Pirarubicin Cisplatin Ifosfamide	Vincristine Doxorubicin Cyclophosphamide Ifosfamide Etoposide Dacarbazine Gemcitabine Docetaxel Iltecan	Vincristine Doxorubicin Cyclophosphamide Ifosfamide Etoposide	Vincristine Doxorubicin Cyclophosphamide Ifosfamide Etoposide Dacarbazine Gemcitabine Docetaxel
	Radiotherapy	Yes	No	No	Yes (proton)	No	Yes
	Targeted therapy	Anlotinib Pazopanib Lenvatinib	No	Anlotinib	Anlotinib Pazopanib	No	Anlotinib Pazopanib Apatinib
	Immunotherapy	Toripalimab	No	No	No	No	No
Prognosis	Current state	DOD	DOD	LWD	DOD	DOD	DOD
	Time of OS	21	3	9	46	12	20

DOD = die of disease, IHC = immunohistochemistry, LWD = live with disease, MyoD1 = myogenic differentiation 1, OS = overall survival.

Case 1 was diagnosed with a malignant peripheral nerve sheath tumor with rhabdomyoblast differentiation, commonly referred to as a salamander tumor. Case 3 presented as spindle cell sarcoma, which could not be further classified. Case 6 was diagnosed with synovial sarcoma (Table 1).

**Figure 2.**
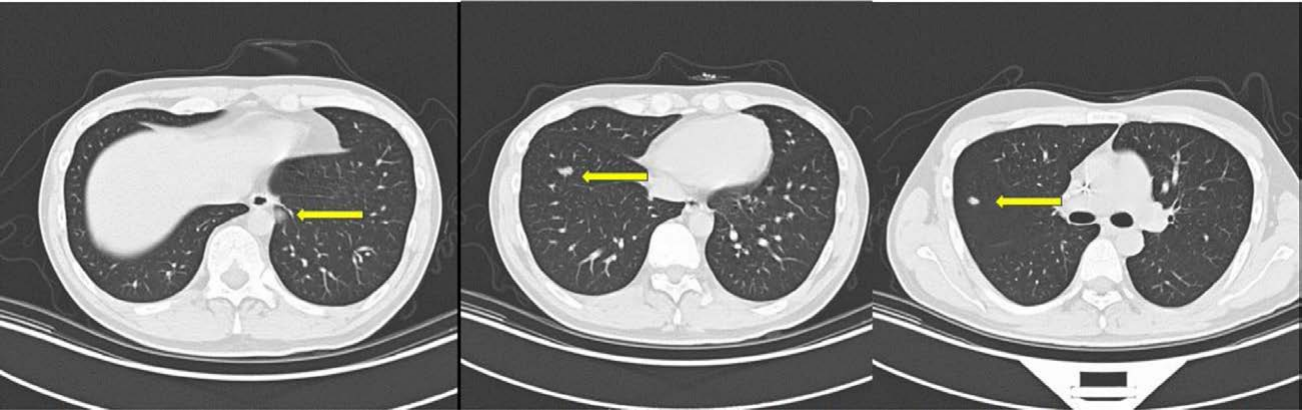
Chest CT of Case 4. CT = computed tomography.

Cases 1, 3, and 5 all exhibited vascular tumor thrombi, while the pathological results for Cases 2, 4, and 6 did not provide information regarding vascular invasion. The Ki-67 expression levels in the tumors of the 6 patients were all ≥70%, with Case 5 exhibiting an expression level of 85%. Case 2 underwent a total cystectomy followed by ileal bladder replacement surgery; however, multiple metastases were detected in the pelvic and abdominal wall 1 month post-operation. Case 3 initially had a biopsy performed on the prostate mass. After obtaining the pathological diagnosis, neoadjuvant chemotherapy was administered. Following chemotherapy, a retropubic radical prostatectomy was conducted. A positron emission tomography/computed tomography scan conducted 2 months after the surgery revealed multiple metastases in the pelvic wall, perineum, perianal region, anterior lower abdominal wall on both sides, as well as bone metastases in both lungs and the body, along with tumor infiltration in the posterior urethra and lower rectum. Case 4 underwent a transurethral electroresection biopsy in Japan. After the pathological diagnosis, neoadjuvant chemotherapy consisting of vincristine, pirarubicin, and cyclophosphamide (VAC regimen) was administered for 6 cycles. Following chemotherapy, a Da Vinci R0 resection of the prostate sarcoma was performed. Postoperative proton radiotherapy was delivered at a dose of 45 Gray over 25 fractions. After completing radiotherapy, VAC chemotherapy was resumed. Upon reexamination after 4 rounds of postoperative chemotherapy, multiple metastases in both lungs were identified (Table 1; Fig. 2).

**Figure 3.**
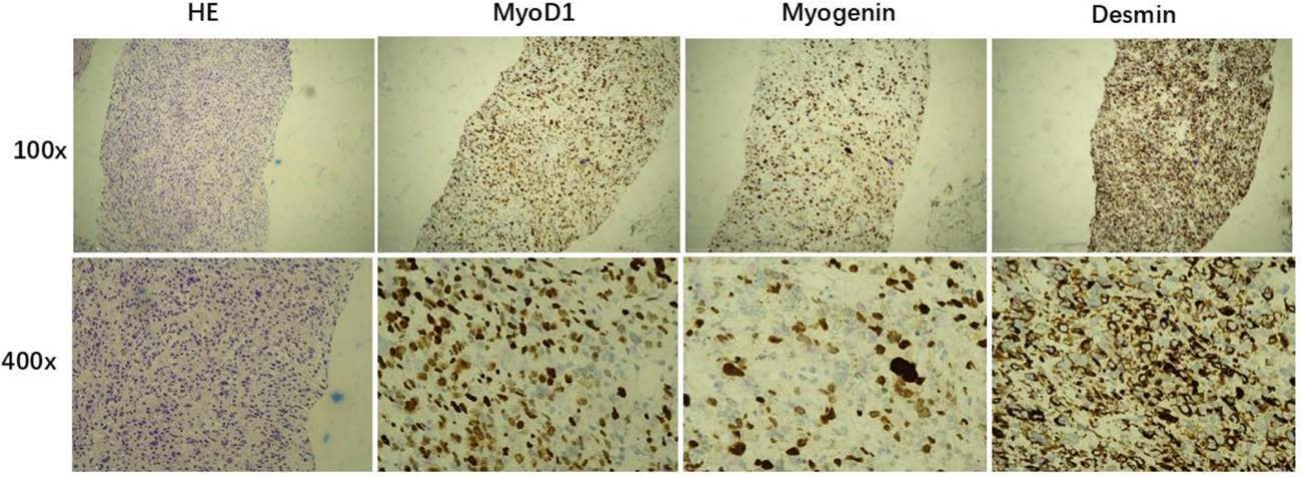
The HE staining and the IHC of positive markers MyoD1, myogenin and desmin of completely embryonal rhabdomyosarcoma. HE = hematoxylin-esion staining, IHC = immunohistochemistry, MyoD1 = myogenic differentiation 1.

All 6 patients received first-line chemotherapy with anthracyclines and ifosfamide. Case 1 also included dacarbazine in their treatment regimen, while Case 2 received vincristine and cisplatin. Following the failure of the first-line regimen, which involved alternating chemotherapy with VAC and ifosfamide + etoposide for Cases 4 and 5, second-line chemotherapy was administered, comprising dacarbazine, gemcitabine, docetaxel, and irinotecan. With the exception of Cases 2 and 4, the remaining 4 patients were treated with anlotinib targeted therapy. After the failure of anlotinib, Cases 2, 4, and 6 were subsequently treated with pazopanib. Following the unsuccessful treatment with pazopanib, Case 1 was administered lenvatinib, while Case 6 received apatinib. Additionally, Case 1 underwent toripalimab immunotherapy, whereas the other 5 patients did not receive any form of immunotherapy (Table 1).

At the time of the last follow-up, only Case 3 was still living with the disease; the remaining 5 patients had succumbed to their illness. Notably, Case 4 had the longest survival duration at 46 months, while Case 3 had the shortest at just 3 months (Table 1).

## 3. Discussion

Primary prostate sarcoma is a rare malignancy of the prostate with a poor prognosis.^[4,5]^ It originates from the mesoderm of the reproductive tract, and its risk factors may be associated with prostatitis, perineal trauma, previous prostate biopsy, and radiation exposure.^[6]^ The main histological types of prostate sarcoma in adults are leiomyosarcoma and rhabdomyosarcoma malignant fibrous histiocytoma, and unclassified sarcoma.^[2,7]^ Among the 6 patients in this report, 3 patients were completely embryonic rhabdomyosarcoma, 1 patient was synovial sarcoma, and 2 patients were other pathological types of sarcoma (Table 1; Fig. 3).

There are no specific presentations of primary prostate sarcoma patients typically report dysuria, urinary retention, and hematuria in the late stages, but often exhibit no symptoms during the early stages. Due to the absence of typical clinical symptoms, the tumor is frequently overlooked or misdiagnosed as benign prostatic hyperplasia. Consequently, in many cases, the prostate is significantly enlarged by the time the tumor is discovered.^[5,8]^ For instance, in Case 6, the tumor measured 4.6 × 7.6 × 3.4 cm, occupying the majority of the prostate, which indicates that the patient is in a serious condition. Therefore, early detection is vital for patients with primary prostate sarcoma. When a tumor reaches a significant size, it can easily invade adjacent organs and tissues. The most extensive invasions often involve the rectum and bladder, leading to symptoms such as abdominal pain and frequent urination. Even in the early stages, primary prostate cancer is highly susceptible to metastasis, typically presenting with multiple metastatic sites at the time of diagnosis.^[9–11]^ In this report, all 4 patients (Case 1, Case 2, Case 5, and Case 6) exhibited pelvic metastases upon initial diagnosis. Additionally, Case 1 presented with metastases to the lungs, liver, and multiple bones. Cases 3 and 4 also developed distant metastasis shortly after surgery. The incidence of lymph node metastasis in primary prostatic sarcoma is low, and no lymph node metastasis was observed in the 6 patients reported here. Patients with primary prostatic sarcoma typically do not exhibit significantly elevated PSA levels, often remaining within the normal reference range. However, the expression of Ki-67 in these tumors is significantly increased, indicating a higher degree of malignancy and a more rapid growth and progression.^[12]^ Prostate carcinoma used to appear mostly in the aged people with obviously increased prostate specific antigen (PSA), while primary prostate sarcoma mostly in younger age with normal PSA, simply because PSA is produced by prostate epithelial cells, while sarcoma originates from stromal cells.

Ultrasound, computed tomography, and magnetic resonance imaging are employed to differentiate prostatic sarcoma from other prostate disorders such as prostate cancer and benign prostatic hyperplasia. However, a definitive diagnosis necessitates pathological evaluation.

The treatment of primary prostate sarcoma primarily involves the management of sarcoma. If surgical intervention is indicated, it should be performed as promptly as possible.^[13]^ Chemotherapy typically relies on doxorubicin and ifosfamide, with first-line regimens often including vincristine, cyclophosphamide, and etoposide.^[14,15]^ Second-line chemotherapy options may also encompass agents such as docetaxel, gemcitabine, and irinotecan.^[16]^ In cases where chemotherapy alone is ineffective, the addition of antiangiogenic agents may be considered, and immunotherapy could also be an option if the patient consents. However, the prognosis for patients, regardless of surgical intervention, remains very poor.

But a multicenter cohort study and comparison between Chinese and American cases showed the status of negative distant metastasis (*P* = .004) and radical tumor resection with negative margin (*P* = .001) were significantly associated with better overall survival (OS), whereas age, tumor size, duration of initial symptoms, and chemo/radiotherapy were not significantly related to OS. The survival time was longer in patients with rhabdomyosarcoma than in those with leiomyosarcoma (*P* = .049).^[17]^ Carolin Siech et al conducted a statistical analysis of the epidemiology, treatment patterns and survival analysis of adult prostate sarcoma. The results showed that the most common pathological types are s leiomyosarcoma, rhabdomyosarcoma, stromal sarcoma and sarcoma not otherwise specified. The OS and mortality of patients are closely related to metastasis. Among all histological subtypes, the overall mortality of stromal sarcoma is lower.^[18]^

## 4. Conclusion

This report concludes that adult primary prostate sarcoma is a highly malignant tumor characterized by a lack of specific symptoms in its early stages, making it difficult to detect. The tumor is prone to local infiltration and distant metastasis. Despite the administration of early surgical interventions alongside chemotherapy, radiotherapy, targeted therapy, and immunotherapy, the prognosis for the patient remains unfavorable, ultimately leading to death due to disease progression. Finally, it must be emphasized that for patients with a prostate mass and normal serum PSA who are younger (<50 years old), clinicians should be reminded of the possibility of sarcoma.

## Author contributions

**Conceptualization:** Li Wang.

**Data curation:** Ping Yang.

**Formal analysis:** Ping Yang.

**Funding acquisition:** Yang He.

**Resources:** Yang He.

**Writing – original draft:** Ping Yang.

**Writing – review & editing:** Li Wang.
